# Sepsis-3 definitions predict ICU mortality in a low–middle-income country

**DOI:** 10.1186/s13613-016-0204-y

**Published:** 2016-11-02

**Authors:** Bruno Adler Maccagnan Pinheiro Besen, Thiago Gomes Romano, Antonio Paulo Nassar, Leandro Utino Taniguchi, Luciano Cesar Pontes Azevedo, Pedro Vitale Mendes, Fernando Godinho Zampieri, Marcelo Park

**Affiliations:** 1 Intensive Care Unit, Emergency Department, Hospital das Clínicas, University of São Paulo Medical School, Rua Dr. Enéas Carvalho de Aguiar, 255, Room 6040, São Paulo, ZIP 05403-000 Brazil; 2 Hospital da Luz, Amil, São Paulo, Brazil; 3 Research Institute, Hospital Sírio-Libanês, São Paulo, Brazil; 4 Nephrology Department, ABC Medical School, Santo Andre, Brazil; 5 A.C. Camargo Cancer Center, São Paulo, Brazil; 6 Research Institute, HCor-Hospital do Coração, São Paulo, Brazil; 7 Intensive Care Unit, Hospital Alemão Oswaldo Cruz, São Paulo, Brazil

**Keywords:** Infection, Intensive care unit, Sepsis, Septic shock, Lactate, Organ dysfunction

## Abstract

**Background:**

Sepsis-3 definitions were published recently and validated only in high-income countries. The aim of this study was to assess the new criteria’s accuracy in stratifying mortality as compared to its predecessor (Sepsis-2) in a Brazilian public intensive care unit (ICU) and to investigate whether the addition of lactate values would improve stratification.

**Methods:**

Retrospective cohort study conducted between 2010 and 2015 in a public university’s 19-bed ICU. Data from patients admitted to the ICU with sepsis were retrieved from a prospectively collected database. ICU mortality was compared across categories of both Sepsis-2 definitions (sepsis, severe sepsis and septic shock) and Sepsis-3 definitions (infection, sepsis and septic shock). Area under the receiving operator characteristic curves were constructed, and the net reclassification index and integrated discrimination index for the addition of lactate as a categorical variable to each stratum of definition were evaluated.

**Results:**

The medical records of 957 patients were retrieved from a prospectively collected database. Mean age was 52 ± 19 years, median SAPS 3 was 65 [50,79], respiratory tract infection was the most common cause (42%, 402 patients), and 311 (32%) patients died in ICU. The ICU mortality rate was progressively higher across categories of sepsis as defined by the Sepsis-3 consensus: infection with no organ dysfunction—7/103 (7%); sepsis—106/419 (25%); and septic shock—198/435 (46%) (*P* < 0.001). For Sepsis-2 definitions, ICU mortality was different only across the categories of severe sepsis [43/252-(17%)] and septic shock [250/572-(44%)] (*P* < 0.001); sepsis had a mortality of 18/135-(13%) (*P* = 0.430 vs. severe sepsis). When combined with lactate, the definitions’ accuracy in stratifying ICU mortality only improved with lactate levels above 4 mmol/L. This improvement occurred in the severe sepsis and septic shock groups (Sepsis-2) and the no-dysfunction and septic shock groups (Sepsis-3). Multivariate analysis demonstrated similar findings.

**Conclusions:**

In a Brazilian ICU, the new Sepsis-3 definitions were accurate in stratifying mortality and were superior to the previous definitions. We also observed that the new definitions’ accuracy improved progressively with severity. Serum lactate improved accuracy for values higher than 4 mmol/L in the no-dysfunction and septic shock groups.

**Electronic supplementary material:**

The online version of this article (doi:10.1186/s13613-016-0204-y) contains supplementary material, which is available to authorized users.

## Background

Sepsis is a well-recognized worldwide healthcare issue, ultimately resulting in significant mortality [1], morbidity and resource utilization during and after critical illness [2]. Initial consensus definitions relied upon the systemic inflammatory response syndrome (SIRS) to infection as a fundamental aspect of sepsis definition, which was then stratified in severity according to the presence of organ dysfunction (severe sepsis) or vasopressor requirement despite adequate fluid resuscitation (septic shock) [3]. This had an important role in education and the pathophysiological understanding of the transition between a homeostatic and a dys-homeostatic inflammatory response that results in organ dysfunctions.

In a second consensus conference, although a new panel of specialists agreed that SIRS was not necessary to the definition of sepsis, not much was changed. The panel recognized several factors that could be used to identify patients with severe sepsis and associated with increased mortality, ultimately leading to important efforts for the early recognition and treatment of severe sepsis and septic shock. This consensus also developed the PIRO concept (predisposition, infection, response, organ failure); however, this idea was not largely translated to clinical practice [4].

Sepsis definitions based on SIRS criteria have, though, been questioned as of late, as SIRS has been shown to be present in 93% of all patients admitted to intensive care (and therefore overly non-specific) [5]. Furthermore, up to 1 in every 8 patients with infection and organ dysfunction do not meet SIRS criteria [6]. Moreover, the methodology used to capture SIRS criteria may lead to substantial variability in defining sepsis cases [7, 8]. These and other concerns have led to the development and publication of new sepsis definitions (Sepsis-3), which were derived through a data-driven mortality risk stratification [9–11]. Although these new criteria have been validated in large databases, much controversy still surrounds them. Some criticisms include the new definitions’ lack of validation in scenarios outside of high-income countries, and their non-utilization of lactate as a marker of organ dysfunction [12–16].

Therefore, in order to address these concerns, we aimed to evaluate whether the new Sepsis-3 definitions maintain their accuracy in a different setting from those in which the criteria were developed and whether they can discriminate intensive care unit (ICU) mortality better than the previous definitions (Sepsis-2). We also evaluated whether the addition of lactate to the definitions would improve upon the previous and new criteria’s accuracy of ICU mortality discrimination.

## Methods

### Study design, population and ethical requirements

This was a retrospective cohort study of patients diagnosed with sepsis admitted to an ICU over a 6-year span, from January 2010 to December 2015. Data were retrieved from the prospectively collected database of a 19-bed ICU in a Brazilian, academic, tertiary medical center in São Paulo—Brazil (Hospital das Clínicas, University of São Paulo Medical School). The study protocol followed the tenets of the Declaration of Helsinki. The institutional review board (*Comissão para Análise de Projetos de* Pesquisa—CAPPesq) reviewed and approved this study (CAPPesq—protocol number 107.443). Since the study was retrospective in nature and did not involve patient identification, informed consent was waived as there was no intervention.

### Setting and sepsis management

Our ICU is located in the Hospital das Clínicas, the largest healthcare complex in Latin America. At the time when the study was conducted, it consisted of seven specialized institutes, with a total of 2400 beds. In the Instituto Central, there are 7 ICUs, ours being mainly a Medical ICU, with patients from emergency surgery and trauma being admitted occasionally for logistical reasons.

The ICU is managed by staff as follows: one nursing assistant for every two beds; one nurse for every five beds; one respiratory therapist for every 10 beds; one staff physician for every 5–8 beds; and residents of internal medicine, critical care, physical therapy, nutrition and nursing. The studied ICU is classified as a strained unit as occupation rate has been above 95% since the beginning of the study period, and the mean SAPS 3 of the patients is 60 [17]. Neither quantitative resuscitation nor protocolized care is routinely used for sepsis management. Our approach to sepsis care is described in Additional file 1.

### Data collection

Our database is an electronic health chart record fulfilled by physicians and respiratory therapists on a daily basis. Patients were selected using *sepsis*, *severe sepsis* or *septic shock* as an [All field] search term in the syndromic diagnosis fields of the database. Senior intensivists clinically adjudicated these diagnoses based on previous Sepsis-2 consensus. Patients with any acute organ failure were considered to have severe sepsis, while septic shock was defined when patients were on vasopressors despite fluid resuscitation. These were adjudicated independent of the presence of 2 SIRS criteria. The following clinical data were collected: age; gender; worst and best vital signs during the first ICU day; Simplified Acute Physiological Score (SAPS) 3; first day total Sequential Organ Failure Assessment (SOFA) score; syndromic diagnosis; etiological diagnosis; comorbidities; ICU length of stay (LOS); organ support measures; clinical ICU outcomes; and highest lactate level of the first day. Laboratory variables were retrieved from the electronic health database specific to laboratorial data.

### Sepsis definitions

Sepsis-2 definitions were primarily used to classify our patients in the database. The categories (sepsis, severe sepsis and septic shock) were defined according to previously published consensus [4]. The new Sepsis-3 categories were defined as follows [9]:

- *Infection without acute organ dysfunction (no-dysfunction category)* infected patients with no significant additional organ dysfunction over the previous conditions, that is, a variation of total SOFA <2 over baseline (chronic organ dysfunction) during the first 24 h after ICU admission.
- *Sepsis (sepsis category)* infected patients with a total SOFA variation ≥2 over baseline clinical condition.
- *Septic shock (septic shock category)* infected patients with persistent hypotension [mean arterial blood pressure (MAP) <65 mmHg] after adequate fluid resuscitation needing vasopressors to keep MAP ≥65 mmHg. Additionally, the hypotension or need for vasopressors must be associated with lactate level >2 mmol/L measured during the first 24 h.

Baseline total SOFA score was considered to be 4 in patients undergoing chronic dialysis, and 2 or 3 in cirrhotic patients, depending on baseline bilirubin levels. For instance, according to the Sepsis-3 definition [9], a patient with chronic renal failure undergoing dialysis was considered to be in the sepsis group only when the total SOFA score was ≥6. Although the original validation of Sepsis-3 definitions did not include patients with ICU LOS <2 days [9], we included all patients in the period described, independently of LOS, in order to pragmatically test the definitions in our ICU.

### Outcome

The primary outcome of interest was ICU mortality.

### Statistical analysis

Continuous variables were assessed for normality using the Kolmogorov–Smirnov test. Parametric continuous variables were compared between groups using the *t* test; nonparametric variables were compared using the Mann–Whitney test. Categorical variables were compared between groups using the Chi-squared test.

We assessed the independent association between lactate and outcome regardless of definition (both Sepsis-2 and Sepsis-3) group using a logistic regression model. Six models were built, three for each definition considering three different cutoffs for lactate (as a continuous predictor, stratified at ≤2 or >2 mEq/L and at ≤4 and >4 mEq/L). Since serum lactate measurements were positively skewed, we used log transformation to fit it into the model as a continuous variable. We further assessed the independent association of Sepsis-3 category and lactate level after adjusting for age, severity at baseline (SAPS 3) and use of support measures (renal replacement therapy, mechanical ventilation). Demographic variables (age and sex) were selected based on univariate analysis, with p values < 0.2 used as cutoff. The other confounding variables were selected because of clinical relevance.

The effect of adding lactate (as a continuous variable or stratified at ≤2 or >2 mEq/L or at ≤4 or >4 mEq/L) on model performance was assessed through area under receiver operating characteristic curve (AUROC). AUROCs were compared using DeLong’s test. Additionally, we assessed the impact of adding lactate to the model using continuous net reclassification improvement (NRI) and integrated discrimination index (IDI), as previously suggested [18]. NRI compares classifications from 2 models (in this case, with and without lactate) for changes by outcome for a net calculation of changes in the right correction. IDI integrates the NRI over all possible cutoffs and is equivalent to the difference in discrimination slopes. In this paper, based on the histograms of the probability of ICU mortality distributions, the cutoff values for reclassification were the following: <30; 30–60; and >60%. We do not report categorical NRI since no single probability cutoff has been defined for the decision-making process in caring for septic patients, as discussed in the Sepsis-3 consensus [10].

Missing values were handled as previously suggested for studies in critical care, when missing values are <5% [19]. There were 46 (<5%) missing lactate values of the first day, which were replaced by the first second day value, or by median imputation if there were not data available. All statistical analyses and graphs were done in R-free source software [20].

## Results

The general characteristics of patients, as well as support measures, clinical outcomes, source of infection, and comorbidities are given in Table 1. For the whole group, mean age was 52 years, without gender differences. The most frequent infectious source was respiratory, followed by abdominal and urinary tract sources. The most frequent comorbidities were diabetes, heart failure and hypertension. Sixty percentage of patients used vasopressors; 44%, mechanical ventilation; and 24%, renal replacement therapy. ICU mortality was 32%. In univariate analysis, non-survivors had higher mean age, severity at admission (SAPS 3), more organ dysfunctions (SOFA) and higher worst lactate values. No infectious source was associated with mortality, neither comorbidities, except for hematological cancer. They also presented more frequently with septic shock when compared to other categories. Furthermore, invasive support measures were more frequently used in this population.

**Table 1:** General characteristics, support measures and outcomes of patients

Variables	Whole group (*N* = 957)	Survivors (*N* = 646)	Non-survivors (*N* = 311)	*P* value^a^
*General characteristics*				
Age—yo	52 ± 19	50 ± 19	56 ± 18	<0.001
Gender (males/females)—*n*	504/453	331/315	173/138	0.291
SAPS 3	65 [50,79]	60 [48,72]	74 [59,90]	<0.001
Total SOFA (first day)	6 [3,9]	5 [3,8]	9 [6,12]	<0.001
Worst lactate (first day)—mmol/L	3.0 [2.0,4.0]	2.7 [1.9,3.7]	3.7 [2.2,5.8]	<0.001
Maximum SOFA (ICU stay)	7 [6,12]	6 [5,9]	12 [8,16]	<0.001
*Support measures*				
Vasopressors—*n* (%)	572 (60)	322 (50)	250 (80)	<0.001
Inotropes—*n* (%)	13 (1)	11 (2)	2 (1)	<0.001
Mechanical ventilation (first day)—*n* (%)	259 (27)	131 (20)	128 (41)	<0.001
(C)RRT (first day)—*n* (%)	102 (11)	51 (8)	51 (16)	<0.001
Mechanical ventilation (ICU stay)—*n* (%)	420 (44)	267 (41)	153 (49)	0.026
(C)RRT (ICU stay)—*n* (%)	233 (24)	137 (21)	96 (31)	0.001
*Sepsis-2 classification*				
Sepsis—*n* (%)	134 (14)	116 (18)	18 (6)	<0.001
Severe sepsis—*n* (%)	251 (26)	208 (32)	43 (14)	<0.001
Septic shock—*n* (%)	572 (60)	322 (50)	250 (80)	<0.001
*Sepsis-3 classification*				
No-dysfunction—*n* (%)	103 (11)	96 (15)	7 (2)	<0.001
Sepsis—*n* (%)	419 (44)	313 (48)	106 (34)	<0.001
Septic shock—*n* (%)	435 (45)	237 (37)	198 (64)	<0.001
*Infection source*				
Respiratory—*n* (%)	402 (42)	263 (41)	139 (45)	0.271
Abdominal—*n* (%)	107 (11)	75 (12)	32 (10)	0.619
Urinary tract—*n* (%)	84 (9)	63 (10)	21 (7)	0.157
Skin and soft tissue—*n* (%)	72 (8)	45 (7)	27 (9)	0.417
Febrile neutropenia syndrome—*n* (%)	32 (3)	19 (3)	13 (4)	0.420
Catheter related—*n* (%)	22 (2)	17 (3)	5 (2)	0.448
Primary bloodstream infection—*n* (%)	17 (2)	13 (2)	4 (1)	0.592
Osteoarticular—*n* (%)	17 (2)	10 (2)	7 (2)	0.610
Unidentifiable source—*n* (%)	167 (17)	117 (18)	50 (16)	0.493
Others^b^—*n* (%)	37 (4)	24 (4)	13 (4)	1.000
*Comorbidities*				
Diabetes mellitus—*n* (%)	196 (20)	124 (19)	72 (23)	0.174
Chronic heart failure—*n* (%)	107 (11)	71 (11)	36 (12)	0.860
Chronic arterial hypertension—*n* (%)	95 (10)	61 (9)	34 (11)	0.534
Dialysis dependent chronic renal failure—*n* (%)	66 (7)	48 (7)	18 (6)	0.429
Rheumatologic diseases—*n* (%)	59 (6)	37 (6)	22 (7)	0.497
Chronic obstructive pulmonary disease—*n* (%)	38 (4)	25 (4)	13 (4)	0.949
Hematologic cancer—*n* (%)	30 (3)	11 (2)	19 (6)	<0.001
Solid cancer—*n* (%)	26 (3)	17 (3)	9 (3)	0.977
Organ transplantation—*n* (%)	20 (2)	14 (2)	6 (2)	1.000
AIDS^c^—*n* (%)	19 (2)	12 (2)	7 (2)	0.867
Liver cirrhosis—*n* (%)	14 (1)	9 (1)	5 (2)	1.000
*Outcomes*				
Days on mechanical ventilation^d^	3 [1,6]	3 [1,6]	3 [1,4]	0.716
Days on (C)RRT^d^	3 [2,7]	3 [2,7]	3 [2,6]	0.181
Days on vasopressors^d^	2 [1,4]	2 [1,4]	2 [1,4]	0.991
ICU Length of stay—days	5 [4,11]	5 [4,10]	6 [3,13]	0.994
ICU mortality—*n* (%)	311 (32)	0 (0)	311 (100)	na
Death within the first 48 h of ICU—*n* (%)	78 (8)	0 (0)	78 (25)	na
Discharge within the first 48 h of ICU—*n* (%)	85 (9)	85 (13)	0 (0)	na

^a^Comparison between survivors and non-survivors

^b^Others are endocarditis, meningitis, leptospirosis and mediastinitis

^c^AIDS denotes acquired immunodeficiency syndrome

^d^Results using only patients which needed the support. na denotes not applicable

**Fig. 1:**
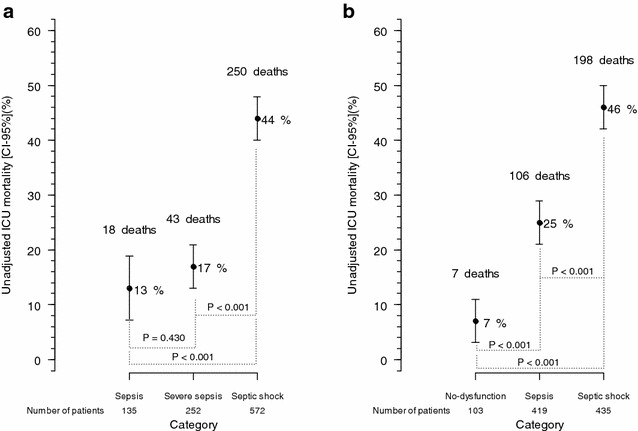
Mortality stratified according to sepsis definition. **a** Mortality according to the Sepsis-2 definition (Pearson’s Chi-squared test using the three categories *P* < 0.001. Pearson’s Chi-squared post hoc analyses are shown in the figure). **b** Mortality according to the Sepsis-3 definition (Pearson’s Chi-squared test using the three categories *P* < 0.001. Pearson’s Chi-squared post hoc analyses are shown in the figure)

Figure 1 depicts that Sepsis-2 definitions show reasonable differentiation in mortality only between septic shock and severe sepsis patients, while Sepsis-3 definitions show clear stratification of mortality among the three categories (no-dysfunction, sepsis and septic shock). Additional file 2: Table 1s shows patients’ re-allocation between both sepsis definitions. Sepsis-3 classification reduced the percentage of septic shock patients as compared to the previous definition.

Additional file 3: Figure 1s shows the median lactate levels of each sepsis category in both sepsis definitions. Significant differences between lactate levels occurred only in the septic shock categories for both definitions (Panels A and C) and between survivors and non-survivors in both septic shock definitions (Panels B and D). The mortality rates according to the sepsis definitions, sepsis categories and serum lactate concentrations are shown in Additional file 4: Fig. 2s. Lactate levels higher than 2 mmol/L were not clearly discriminative of mortality in the Sepsis-2 defined categories of severe sepsis or septic shock. In comparison, lactate levels >4 mmol/L clearly marked increased mortality in both the severe sepsis and septic shock categories of Sepsis-2 and in the no-dysfunction and septic shock categories of Sepsis-3.

 Table 2 shows the associations of sepsis definitions with mortality and the additional impact of lactate inclusion in the multivariate model. Furthermore, Table 3 shows the association of Sepsis-3 definition with ICU mortality, after adjustment for lactate level, age, illness severity (assessed through SAPS 3), requirement for RRT and mechanical ventilation at any time of ICU stay.

**Table 2:** Multivariate models using ICU death as a dependent variable and sepsis definition and lactate as independent variables

	Lactate as a continuous variable		Lactate > 2 mmol/L		Lactate > 4 mmol/L	
	OR (CI 95%)	*P* value	OR (CI 95%)	*P* value	OR (CI 95%)	*P* value
*Sepsis-2 model*						
Sepsis	1	Reference	1	Reference	1	Reference
Severe sepsis	1.06 (0.97–1.16)	0.322	1.03 (1.01–1.19)	0.500	1.04 (0.95–1.19)	0.342
Septic shock	1.34 (1.22–1.44)	<0.001	1.33 (1.22–1.45)	0.001	1.32 (1.21–1.43)	0.001
Lactate	1.02 (1.02–1.03)	0.001	1.07 (1.00–1.14)	0.027	1.21 (1.14–1.30)	0.001
*Sepsis-3 model*						
No-dysfunction	1	Reference	1	Reference	1	Reference
Sepsis	1.21 (1.11–1.34)	<0.001	1.19 (1.08–1.32)	<0.001	1.21 (1.10–1.14)	<0.001
Septic shock	1.41 (1.28–1.56)	<0.001	1.49 (1.35–1.65)	<0.001	1.41 (1.28–1.56)	<0.001
Lactate	1.02 (1.01–1.03)	<0.001	0.97 (0.90–1.05)	0.466	1.19 (1.11–1.28)	<0.001

This analysis was performed using a binary logistic regression. The first model used lactate as a continuous variable (after logarithmic transformation in order to correct the positive skewness); the second and third models used lactate as a categorical variable

Lactate > 2 mmol/L represents all patients with lactate > 2 mmol/L, including those with lactate > 4 mmol/L

**Table 3:** Multivariate models using ICU death as a dependent variable and Sepsis-3 definition, lactate, age, SAPS 3 and requirement for RRT and mechanical ventilation as independent variables

	Lactate as a continuous variable		Lactate > 2 mmol/L		Lactate > 4 mmol/L	
	OR (CI 95%)	*P* value	OR (CI 95%)	*P* value	OR (CI 95%)	*P* value
*Sepsis-3 model*						
No-dysfunction	1	Reference	1	Reference	1	Reference
Sepsis	3.70 (1.75–9.12)	0.002	3.35 (1.57–8.27)	0.004	3.67 (1.73–9.07)	0.002
Septic shock	6.21 (2.94–15.27)	<0.001	7.31 (3.41–18.22)	<0.001	6.22 (2.94–15.31)	<0.001
Lactate	1.11 (1.06–1.16)	<0.001	0.94 (0.60–1.48)	0.811	2.28 (1.61–3.24)	<0.001
Age	1.01 (1.01–1.02)	<0.001	1.01 (1.01–1.02)	0.002	1.01 (1.00–1.02)	<0.001
SAPS 3	1.02 (1.01–1.03)	<0.001	1.02 (1.01–1.03)	<0.001	1.02 (1.01–1.03)	<0.001
RRT^a^	1.63 (1.15–2.31)	0.006	1.56 (1.11–2.21)	0.011	1.66 (1.17–2.36)	0.004
Mechanical ventilation^a^	1.29 (0.95–1.76)	0.107	1.26 (0.92–1.71)	0.146	1.27 (0.93–1.73)	0.131

	AUC^b^ (CI 95%)	Asymptotic *P* value	AUC^b^ (CI 95%)	Asymptotic *P* value	AUC^b^ (CI 95%)	Asymptotic *P* value
Model accuracy	0.732 (0.698–0.766)	<0.001	0.725 (0.691–0.759)	<0.001	0.732 (0.698–0.766)	<0.001

^a^Support needed at any time of ICU stay; *RRT* renal replacement therapy; *SAPS* Simplified Acute Physiological Score

^b^AUC area under the curve

Table 4 shows the accuracy of ICU death prediction of models using only the sepsis definitions and sepsis definitions added to lactate as a continuous and dichotomous variable. The accuracy significantly improved with the addition of lactate to the model.

**Table 4:** Predictive accuracy of mortality of sepsis definitions with or without lactate value

	Sepsis-2 definition		Sepsis-3 definition	
	AUC (CI 95%)	Asymptotic *P* value	AUC (CI 95%)	Asymptotic *P* value
Without lactate model	0.650 (0.619–0.680)	<0.001	0.615 (0.580–0.650)	<0.001
Lactate as a continuous variable^a^	0.704 (0.668–0.739)^b^	<0.001	0.649 (0.608–0.689)^b, d^	<0.001
Lactate > 2 mmol/L	0.664 (0.631–0.697)	<0.001	0.620 (0.584–0.656)	<0.001
Lactate > 4 mmol/L	0.686 (0.653–0.719)^b, c, d^	<0.001	0.636 (0.598–0.674)^b, c, d^	<0.001

The receiver operating curves (ROC) were constructed using the binary logistic regression probabilities of death using sepsis definitions and lactate categories. AUC area under the ROC curve

^a^Lactate as a continuous variable was logarithmically transformed in order to correct the positive skewness. Lactate > 2 mmol/L represents all patients with lactate > 2 mmol/L, including those with lactate > 4 mmol/L

^b^DeLong’s test *P* < 0.05 versus without lactate model

^c^DeLong’s test *P* = ns versus lactate as a continuous variable

^d^DeLong’s test *P* < 0.05 versus lactate > 2 mmol/L

Further evaluation of lactate’s addition to the sepsis definitions model using the net reclassification index (NRI) and integrated discrimination increment (IDI) is presented in Additional file 2: Table 2s. In Sepsis-2 definitions, NRI as it relates to ICU mortality was 23.1 (*P* < 0.001) for lactate >2 mmol/L and 39.9 (*P* < 0.001) for lactate >4 mmol/L. For Sepsis-3 definitions, NRI was 39.9 (*P* < 0.001) for lactate levels >4 mmol/L. The discrimination slope of the updated model with lactate levels >4 mmol/L, as assessed by IDI, was 3.3 (*P* < 0.001) for Sepsis-2 and 2.7 (*P* < 0.001) for Sepsis-3. For lactate levels >2 mmol/L, IDI was 0.7 (*P* = 0.021) for Sepsis-2 criteria, while it was not significant for Sepsis-3.

## Discussion

In this study, we demonstrate that Sepsis-3 is better than Sepsis-2 at stratifying mortality among septic patients admitted in a strained ICU of a developing country. Sepsis-3 criteria clearly categorized septic patients along a spectrum of severity, since mortality increased from 7% in no-dysfunction-infected patients to 25 and 46% for sepsis and septic shock patients, respectively. In contrast, in the Sepsis-2 definitions, only the septic shock criterion was associated with a significantly higher mortality when compared to the severe sepsis and sepsis categories (44, 17 and 13%, respectively). Moreover, a lactate level higher than 4 mmol/L improved the mortality prediction accuracy on both Sepsis-2 and Sepsis-3 definitions (Table 3).

The validity of sepsis definitions based on SIRS criteria has recently been called into question. This is because almost 90% of the patients admitted to an intensive care unit (ICU) meet the SIRS criteria, [5, 21]. Even SIRS criteria’s high sensitivity has been questioned recently, as some patients with known infectious insult and new organ failures do not satisfy 2 SIRS criteria and therefore do not fulfill previous sepsis definitions [6]. Even more importantly, the categories of sepsis, severe sepsis and septic shock should indicate a real spectrum of severity to be considered as different outcome categories [14]. The present study reinforces a lack of accuracy of Sepsis-2 definitions of sepsis, severe sepsis and septic shock in stratifying patients’ mortality risk. Additionally, our data demonstrate Sepsis-3 definitions of infection without organ dysfunction, sepsis and septic shock clearly represent progressive strata of mortality risk (Fig. 1).

High lactate values in sepsis may be the result of perfusion abnormalities or stress hyperlactatemia [22]. Regardless of the reason for its increase, early high lactate value is traditionally associated with worse outcomes in septic [23] and other critically ill patients [24, 25]. The fact that lactate was not maintained as a marker of organ dysfunction in the new sepsis definitions raised several concerns [26, 27], and the Surviving Sepsis Campaign suggested that lactate should continue to be measured to identify dysfunction [27]. In the original validation study of Sepsis-3 definitions, although high lactate levels were associated with higher mortality at different cutoffs regardless of hypotension or vasopressors, lactate was retained only for the characterization of septic shock [9]. In the present study, however, lactate was a prognostic factor for both definitions and reclassified some Sepsis-3-defined patients when its levels were above 4 mmol/L. In the assessment of the new septic shock definitions in the original validation study, lactate levels higher than 2 mmol/L were increasingly associated with higher odds of death [11]. In our study, cutoff levels above 2 mmol/L did not add any prognostic information in this stratum of severity, as expected, because it is included in the definition itself. However, in accordance with the validation paper, higher lactate levels (which we chose to add in the model as >4 mmol/L) were of prognostic significance.

In the validation of clinical criteria for sepsis, unfortunately, there was no clear mention to the added benefit of lactate for patients with SOFA scores <2, possibly because of lactate values missingness in the derivation (about 60%) and validation cohorts (about 90%) [10]. Interestingly, lactate was not retained in the novel qSOFA during model construction. However, the authors state that for a qSOFA score of 1, high lactate values characterized a population with similar risk to patients with a qSOFA score of 2 [10]. In our cohort, for patients without organ dysfunction, lactate levels higher than 4 mmol/L seem to categorize a population at higher risk of death (Table 2 and Additional file 4: Fig. 2s). Therefore, high lactate levels in patients without organ dysfunction as assessed by the SOFA score might improve risk stratification and change management of these patients, since they could be allocated to a higher level of care upfront in order to avoid further deterioration of their clinical status.

Sepsis-3 criteria for identifying septic patients have been extensively questioned since its publication [12, 15]. A possible decrease in awareness of sepsis and a need for massive educational programs to healthcare providers are among the main concerns. In this context, how Sepsis-3 criteria would perform for sepsis detection, especially in countries with high mortality rates, still needs validation. Our study was not designed to assess Sepsis-2 or Sepsis-3 as a screening tool, since only patients admitted to ICU were included. The role of Sepsis-2 criteria for identifying septic patients is undeniable, as was the case in a recent before–after study conducted in Brazil [28]. However, its accuracy to stratify severity can be called into question, as demonstrated by our data. Accordingly, our study clearly shows a better discriminative performance of Sepsis-3 in predicting ICU mortality.

Strengths of this study include the number of patients analyzed, the recent timeframe and the use of statistical methodologies such as net reclassification index, which allowed us to better evaluate the benefit of adding lactate to the new definition. Also, NRI was not used in the original validation study and our data missingness for lactate values was much lower (<5%), which could have yielded some of the above different results. Furthermore, this represents an external assessment of new criteria in a different setting than that of the original validation—an important step toward consistency for its findings.

There are, nevertheless, several limitations to be addressed, in light of which our findings should be interpreted. First, the retrospective nature of this study makes it difficult to elucidate known confounders that could have biased the outcome measures, such as differences in sepsis treatment. Second, this was a single-center study consisting mainly of medical patients and our results may not be generalizable to other centers, including from other developing countries, since there is wide variation in outcomes even within Brazil when comparing different settings [29, 30]. Third, we evaluated only patients admitted to the ICU and, therefore, our findings cannot be extrapolated to patients treated in wards and in the emergency room. This also threatens the generalizability of our results, since the original study captured a population with presumed infection not only in the ICU. Fourth, the validity of Sepsis-3 criteria in this study was assessed based on ICU mortality. Although ICU mortality is not the most appropriate endpoint to be evaluated, it reflects the pre-ICU and ICU care of septic patients, and in our scenario, identified a more specific point to improve the care of septic patients [31, 32].

## Conclusions

The new clinical criteria of sepsis proposed by the third international consensus (Sepsis-3) could predict mortality in infected patients admitted to a strained ICU in a middle-income country. The prognostic value of the new definition of sepsis was progressive along all three categories, which denotes a spectrum of gravity of infectious process and could help in risk stratification for future studies in this area of research. This was not observed of the previous definitions (Sepsis-2) in our sample. Serum lactate improved accuracy for values higher than 4 mmol/L in the no-dysfunction and septic shock groups.

## Additional files

**Additional file 1: Appendix**

**Additional file 2: Tables**

**Additional file 3: Figure 1s** Lactate levels according to the sepsis categories. **Panel A** shows lactate levels according to the Sepsis-2 definition. **Panel B** shows lactate levels according to the Sepsis-2 definition, stratified according to survival. **Panel C** shows lactate levels according to the Sepsis-3 definition. **Panel D** shows lactate levels according to the Sepsis-3 definition, stratified according to survival. * Kruskal-Wallis’ test P < 0.001 among the three sepsis categories. Mann-Whitney’s P < 0.05 *post-hoc* analysis vs. other categories. # Mann-Whitney’s test P < 0.05 vs. survivors.

**Additional file 4: Figure 2s** Mortality according to the sepsis category and lactate concentration. Panel A shows the Sepsis-2 categories. Panel B shows the Sepsis-3 categories. Lactate > 2 mmol/L represents all patients with lactate > 2 mmol/L, including those with lactate > 4 mmol/L* Number of patients in the category. # Pearson’s Chi-squared test P < 0.001 among the three sepsis categories (same lactate level). Pearson’s Chi-squared test P < 0.05 *post-hoc* analysis vs. severe sepsis (same lactate level) category.$ Pearson’s Chi-squared test P < 0.001 among the three sepsis categories (same lactate level). Pearson’s Chi-squared test P < 0.05 *post-hoc* analysis vs. sepsis (same lactate level) category. % Pearson’s Chi-squared test P < 0.001 among the three lactate categories (same sepsis category level). Pearson’s Chi-squared test P < 0.05 *post-hoc* analysis vs. whole group and Lactate > 2 mmol/L (same sepsis category level) categories.

## Abbreviations

ICU: intensive care unit
SOFA: Sequential Organ Failure Assessment
SAPS: Simplified Acute Physiological Score
SIRS: systemic inflammatory response syndrome
LOS: length of stay
IDI: integrated discrimination index
NRI: net reclassification improvement
AUROC: area under the receiving operational characteristics curve

## References

[1] Fleischmann C, Scherag A, Adhikari NK, Hartog CS, Tsaganos T, Schlattmann P (2016). Assessment of global incidence and mortality of hospital-treated sepsis. Current estimates and limitations. Am J Respir Crit Care Med.

[2] Kahn JM, Benson NM, Appleby D, Carson SS, Iwashyna TJ (2010). Long-term acute care hospital utilization after critical illness. JAMA.

[3] Bone RC, Balk RA, Cerra FB, Dellinger RP, Fein AM, Knaus WA (1992). Definitions for sepsis and organ failure and guidelines for the use of innovative therapies in sepsis. The ACCP/SCCM Consensus Conference Committee. American College of Chest Physicians/Society of Critical Care Medicine. Chest.

[4] Levy MM, Fink MP, Marshall JC, Abraham E, Angus D, Cook D (2003). 2001 SCCM/ESICM/ACCP/ATS/SIS international sepsis definitions conference. Intensive Care Med.

[5] Sprung CL, Sakr Y, Vincent JL, Le Gall JR, Reinhart K, Ranieri VM (2006). An evaluation of systemic inflammatory response syndrome signs in the Sepsis Occurrence In Acutely Ill Patients (SOAP) study. Intensive Care Med.

[6] Kaukonen KM, Bailey M, Pilcher D, Cooper DJ, Bellomo R (2015). Systemic inflammatory response syndrome criteria in defining severe sepsis. N Engl J Med.

[7] Klein Klouwenberg PM, Ong DS, Bonten MJ, Cremer OL (2012). Classification of sepsis, severe sepsis and septic shock: the impact of minor variations in data capture and definition of SIRS criteria. Intensive Care Med.

[8] Gaieski DF, Edwards JM, Kallan MJ, Carr BG (2013). Benchmarking the incidence and mortality of severe sepsis in the United States. Crit Care Med.

[9] Singer M, Deutschman CS, Seymour CW, Shankar-Hari M, Annane D, Bauer M (2016). The third international consensus definitions for sepsis and septic shock (sepsis-3). JAMA.

[10] Seymour CW, Liu VX, Iwashyna TJ, Brunkhorst FM, Rea TD, Scherag A (2016). Assessment of clinical criteria for sepsis: for the third international consensus definitions for sepsis and septic Shock (sepsis-3). JAMA.

[11] Shankar-Hari M, Phillips GS, Levy ML, Seymour CW, Liu VX, Deutschman CS (2016). Developing a new definition and assessing new clinical criteria for septic shock: for the third international consensus definitions for sepsis and septic shock (sepsis-3). JAMA.

[12] Cortes-Puch I, Hartog CS (2016). Change is not necessarily progress: revision of the sepsis definition should be based on new scientific insights. Am J Respir Crit Care Med.

[13] Angus DC (2016). Defining sepsis: A case of bounded rationality and fuzzy thinking?. Am J Respir Crit Care Med.

[14] Deutschman CS (2016). Imprecise medicine: the limitations of sepsis-3. Crit Care Med.

[15] Simpson SQ (2016). New sepsis criteria: a change we should not make. Chest.

[16] Vincent JL, Mira JP, Antonelli M (2016). Sepsis: older and newer concepts. Lancet Respir Med.

[17] Gabler NB, Ratcliffe SJ, Wagner J, Asch DA, Rubenfeld GD, Angus DC (2013). Mortality among patients admitted to strained intensive care units. Am J Respir Crit Care Med.

[18] Steyerberg EW, Vickers AJ, Cook NR, Gerds T, Gonen M, Obuchowski N (2010). Assessing the performance of prediction models: a framework for traditional and novel measures. Epidemiology.

[19] Vesin A, Azoulay E, Ruckly S, Vignoud L, Rusinova K, Benoit D (2013). Reporting and handling missing values in clinical studies in intensive care units. Intensive Care Med.

[20] R Core Team. R: A language and environment for statistical computing. R Foundation for Statistical Computing, Vienna, Austria; 2014. http://www.R-project.org/. Accessed 25 May 2016.

[21] Lai NA, Kruger P (2011). The predictive ability of a weighted systemic inflammatory response syndrome score for microbiologically confirmed infection in hospitalized patients with suspected sepsis. Crit Care Resusc.

[22] Van Lambalgen AA, Runge HC, van den Bos GC, Thijs LG (1988). Regional lactate production in early canine endotoxin shock. Am J Physiol Endocrinol Metab.

[23] Noritomi DT, Soriano FG, Kellum JA, Cappi SB, Biselli PJ, Liborio AB (2009). Metabolic acidosis in patients with severe sepsis and septic shock: a longitudinal quantitative study. Crit Care Med.

[24] Shapiro NI, Howell MD, Talmor D, Nathanson LA, Lisbon A, Wolfe RE (2005). Serum lactate as a predictor of mortality in emergency department patients with infection. Ann Emerg Med.

[25] Trzeciak S, Dellinger RP, Chansky ME, Arnold RC, Schorr C, Milcarek B (2007). Serum lactate as a predictor of mortality in patients with infection. Intensive Care Med.

[26] Machado FR, Salomao R, Pontes de Acevedo LC, Lisboa T, Costa Filho R. Latin American Sepsis Institute (LASI). Why LASI did not endorse the new definitions of sepsis published today in JAMA; 2016. http://ilas.org.br/assets/arquivos/upload/declaracao%20sepse%203.0%20ILAS%20-%20English%20version.pdf. Accessed 25 May 2016.

[27] Antonelli M, De Backer D, Dorman T, Kleinpell R, Levy M, Rhodes A. For the Surviving Sepsis Campaign Executive Committee. Surviving Sepsis Campaign Responds to Sepsis-3; 2016. http://www.survivingsepsis.org/SiteCollectionDocuments/SSC-Statements-Sepsis-Definitions-3-2016.pdf. Accessed 25 May 2016.

[28] Noritomi DT, Ranzani OT, Monteiro MB, Ferreira EM, Santos SR, Leibel F (2014). Implementation of a multifaceted sepsis education program in an emerging country setting: clinical outcomes and cost-effectiveness in a long-term follow-up study. Intensive Care Med.

[29] Silva E, Pedro Mde A, Sogayar AC, Mohovic T, Silva CL, Janiszewski M (2004). Brazilian Sepsis Epidemiological Study (BASES study). Crit Care.

[30] Conde KA, Silva E, Silva CO, Ferreira E, Freitas FG, Castro I (2013). Differences in sepsis treatment and outcomes between public and private hospitals in Brazil: a multicenter observational study. PLOS ONE.

[31] Berenholtz SM, Pronovost PJ, Ngo K, Barie PS, Hitt J, Kuti JL (2007). Developing quality measures for sepsis care in the ICU. J Comm J Qual Patient Saf.

[32] Berenholtz SM, Dorman T, Ngo K, Pronovost PJ (2002). Qualitative review of intensive care unit quality indicators. J Crit Care.

