# Supplementary material for: Sepsis-3 definitions predict ICU mortality in a low–middle-income country
Source: Ann Intensive Care. 2016 Nov 2;6:107. doi: 10.1186/s13613-016-0204-y (PMC5093106; doi:10.1186/s13613-016-0204-y)
Supplement: Supplementary file 1 — Additional file 1: Appendix [file 13613_2016_204_MOESM1_ESM.docx]

**Supplementary Appendix**

In this appendix, we provide additional information regarding patients’ care at the intensive care unit and additional tables.

***Patients’ care***

All patients in this cohort received antimicrobials. In this ICU, central venous and arterial catheters are used under clinical judgment.Central venous catheterization is habitually used in circumstances where a peripheral vein is not available or when vasopressors are needed.

Shock resuscitation is initially done with volume challenge of 500 – 1000 mL of lactated Ringer’s if not contraindicated by other clinical factors, such as known severe heart failure, anasarca, jugular engorgement, and severe respiratory failure. After this initial approach, norepinephrine is titrated to maintain a mean arterial pressure ≥ 65 mmHg. Further hemodynamic evaluation is routinely done with body ultrasound (thorax and echocardiography). When possible, echocardiography is used to evaluate the aortic velocity-time-integral (VTI) to calculate cardiac output. Echocardiography is also employed in the evaluation of inferior vena cava to estimate central venous pressure; and also for global evaluation of both left ventricular and right ventricular function. With these parameters, and dynamic indices of fluid responsiveness when applicable, we guide fluid management and titrate inotropes. Lactate is collected upon ICU admission in all patients, and further measurements are taken if clinically indicated. Lactate is not recommended to be used alone as a goal for hemodynamic resuscitation in our unit, but only within the first six hours after shock diagnosis (hypotensive or cryptic) and if a clinical sign of hypoperfusion is evident (capillary refilling time > 4 seconds, mottling, and extremity cyanosis). Central venous oxygen saturation (ScvO_2_) is not recommended for patients’ routine monitoring, unless clinical evidence of low cardiac output is observed through clinical examination or echocardiography. Dobutamine is the inotrope of choice. Epinephrine is occasionally used as an inotrope in patients with refractory shock (norepinephrine > 0.5 mcg/kg/minute). Vasopressin is not used as a first-line vasopressor and is only occasionally indicated incases of vasodilatory septic shock requiring high doses of catecholamines. Corticosteroids are used in patients with raising high-doses of norepinephrine in the absence of contraindications by other clinical factors.
