# Supplementary material for: Sepsis-3 definitions predict ICU mortality in a low–middle-income country
Source: Ann Intensive Care. 2016 Nov 2;6:107. doi: 10.1186/s13613-016-0204-y (PMC5093106; doi:10.1186/s13613-016-0204-y)
Supplement: Supplementary file 2 — Additional file 2: Tables [file 13613_2016_204_MOESM2_ESM.docx]

***Supplementary Tables***

| **Table 1s: Cross tabulation between the two definitions** | | | | | |
| --- | --- | --- | --- | --- | --- |
|  |  |  |  |  |  |
|  |  | Sepsis-2 | | |  |
|  |  | Sepsis | Severe sepsis | Septic shock | Total |
| Sepsis-3 | No-dysfunction | 40 | 63 | 0 | 103 |
|  | Sepsis | 94 | 188 | 137 | 419 |
|  | Septic shock | 0 | 0 | 435 | 435 |
|  | Total | 134 | 251 | 572 | 957 |
|  |  |  |  |  |  |

| **Table 2s: Net Reclassification Index and Integrated Discrimination Increment of lactate’s addition to the sepsis definitions in association with ICU death** | | | | |
| --- | --- | --- | --- | --- |
|  |  |  |  |  |
| Prediction model | Net Reclassification Index  (NRI) (CI-95%) | P value | Integrated Discrimination Increment  (IDI) (CI-95%) | P value |
|  |  |  |  |  |
| Sepsis-2 |  |  |  |  |
|  |  |  |  |  |
| Without lactate | reference |  | reference |  |
| Lactate continuous * | 0.234 (0.105 – 0.364) | < 0.001 | 0.031 (0.018 – 0.364) | < 0.001 |
| Lactate > 2 mmol/L | 0.231 (0.113 – 0.349) | < 0.001 | 0.007 (0.002 – 0.043) | 0.021 |
| Lactate > 4 mmol/L | 0.399 (0.277 – 0.521) | < 0.001 | 0.033 (0.020 – 0.045) | < 0.001 |
|  |  |  |  |  |
| Sepsis-3 |  |  |  |  |
|  |  |  |  |  |
| Without lactate | reference |  | reference |  |
| Lactate continuous * | 0.178 (0.046 – 0.310) | 0.008 | 0.020 (0.010 – 0.029) | < 0.001 |
| Lactate > 2 mmol/L | - 0.231 (-0.349 – - 0.113) | < 0.001 | 0.0005 (- 0.00005 – 0.00008) | 0.690 |
| Lactate > 4 mmol/L | 0.399 (0.277 – 0.521) | < 0.001 | 0.027 (0.016 – 0.038) | < 0.001 |
|  |  |  |  |  |

* In the prediction logistic binary model, lactate was logarithmically transformed to correct the positive skewness.

Lactate > 2 mmol/L represents all patients with lactate > 2 mmol/L, including those with lactate > 4 mmol/L.
