# Supplementary material for: Sepsis-3 definitions predict ICU mortality in a low–middle-income country
Source: Ann Intensive Care. 2016 Nov 2;6:107. doi: 10.1186/s13613-016-0204-y (PMC5093106; doi:10.1186/s13613-016-0204-y)
Supplement: Supplementary file 3 — Additional file 3: Figure 1s Lactate levels according to the sepsis categories. Panel A shows lactate levels according to the Sepsis-2 definition. Panel B shows lactate levels according to the Sepsis-2 definition, stratified according to survival. Panel C shows lactate levels according to the Sepsis-3 definition. Panel D shows lactate levels according to the Sepsis-3 definition, stratified according to survival. * Kruskal-Wallis’ test P < 0.001 among the three sepsis categories. Mann-Whitney’s P < 0.05 post-hoc analysis vs. other categories. # Mann-Whitney’s test P < 0.05 vs. survivors. [file 13613_2016_204_MOESM3_ESM.pdf]

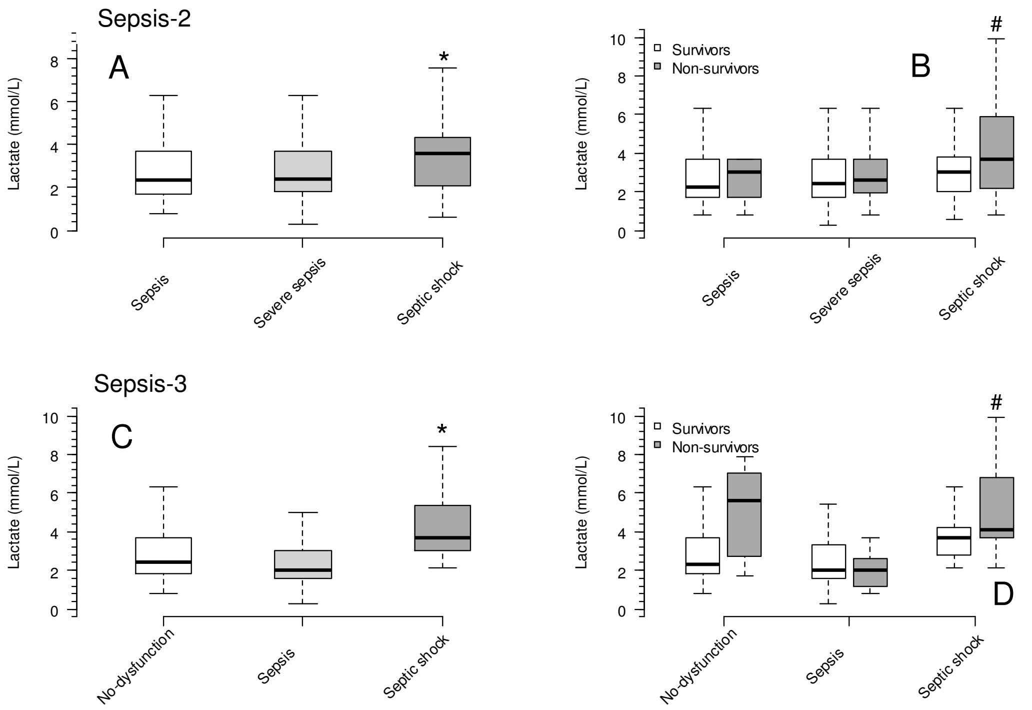

Figure 1s: Lactate levels according to the sepsis categories**. Panel A** shows lactate levels according to the sepsis-2 definition. **Panel B** shows lactate levels according to the sepsis-2 definition, stratified according to the survival. **Panel C** shows lactate levels according to the sepsis-3 definition. **Panel D** shows lactate levels according to the sepsis-3 definition, stratified according to the survival.

* Kruskal-Wallis' test P < 0.001 among the three sepsis categories. Mann-Whitney's P < 0.05 *post-hoc* analysis vs. other categories.

# Mann-Whitney's test P < 0.05 vs. survivors.
