# Supplementary material for: Sepsis-3 definitions predict ICU mortality in a low–middle-income country
Source: Ann Intensive Care. 2016 Nov 2;6:107. doi: 10.1186/s13613-016-0204-y (PMC5093106; doi:10.1186/s13613-016-0204-y)
Supplement: Supplementary file 4 — Additional file 4: Figure 2s Mortality according to the sepsis category and lactate concentration. Panel A shows the Sepsis-2 categories. Panel B shows the Sepsis-3 categories. Lactate > 2 mmol/L represents all patients with lactate > 2 mmol/L, including those with lactate > 4 mmol/L* Number of patients in the category. # Pearson’s Chi-squared test P < 0.001 among the three sepsis categories (same lactate level). Pearson’s Chi-squared test P < 0.05 post-hoc analysis vs. severe sepsis (same lactate level) category.$ Pearson’s Chi-squared test P < 0.001 among the three sepsis categories (same lactate level). Pearson’s Chi-squared test P < 0.05 post-hoc analysis vs. sepsis (same lactate level) category. % Pearson’s Chi-squared test P < 0.001 among the three lactate categories (same sepsis category level). Pearson’s Chi-squared test P < 0.05 post-hoc analysis vs. whole group and Lactate > 2 mmol/L (same sepsis category level) categories. [file 13613_2016_204_MOESM4_ESM.pdf]

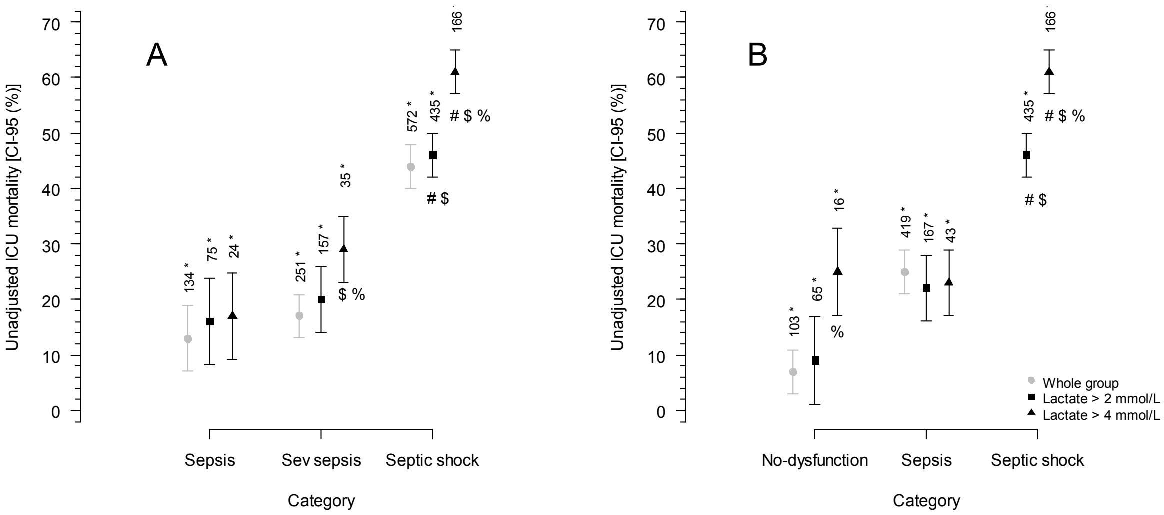

Figure 2s: Mortality according to the sepsis category and lactate concentration. **Panel A** shows the sepsis-2 categories. **Panel B** shows the sepsis-3 categories.

Lactate > 2 mmol/L represents all patients with lactate > 2 mmol/L, including those with lactate > 4 mmol/L

* Number of patients in the category.

# Pearson's Chi-squared test P < 0.001 among the three sepsis categories (same lactate level). Pearson's Chi-squared test P < 0.05 *post-hoc* analysis vs. severe sepsis (same lactate level) category.

$ Pearson's Chi-squared test P < 0.001 among the three sepsis categories (same lactate level). Pearson's Chi-squared test P < 0.05 *post-hoc* analysis vs. sepsis (same lactate level) category.

% Pearson's Chi-squared test P < 0.001 among the three lactate categories (same sepsis category level). Pearson's Chi-squared test P < 0.05 *post-hoc* analysis vs. whole group and Lactate > 2 mmol/L (same sepsis category level) categories.
